# Does Sleep Help Prevent Forgetting Rewarded Memory Representations in Children and Adults?

**DOI:** 10.3389/fpsyg.2018.00924

**Published:** 2018-06-08

**Authors:** Alexander Prehn-Kristensen, Annie Böhmig, Juliane Schult, Anya Pedersen, Christian D. Wiesner, Lioba Baving

**Affiliations:** ^1^Department of Child and Adolescent Psychiatry and Psychotherapy, Centre for Integrative Psychiatry, School of Medicine, Christian-Albrechts-University Kiel, Kiel, Germany; ^2^Department of Psychology, Christian-Albrechts-University Kiel, Kiel, Germany

**Keywords:** memory, reward, sleep, children, adults

## Abstract

Sleep fosters the consolidation of rewarded memory representations in adults. However, sleep and its memory-supporting functions change through healthy development, and it is unclear whether sleep benefits the consolidation of rewarded memory representations in children as it does in adults. Based on previous findings, we expected sleep to benefit the consolidation of rewarded memory representations in children more than it does in adults. For that reason, 16 children (7–11 years) and 20 adults (21–29 years) participated in this experiment. During the encoding session, participants were asked to learn the location of 18 object pairs. Thereafter, one-half of the object locations were allocated to a high-rewarded condition and the other half to a low-rewarded condition. In the sleep condition, the encoding session took place in the evening (for children 7–8 pm, for adults 8–9 pm). After a fixed retention interval of 12 h the retrieval session was conducted the next morning (for children 7–8 am, for adults 8–9 am). In the wake condition, the time schedule was the same but reversed: the encoding session started in the morning (for children 7–8 am, for adults 8–9 am), and retrieval took place in the evening (for children 7–8 pm, for adults 8–9 pm). Sleep/wake had no impact on the memory performance regarding the low-rewarded memory items. In contrast, wakefulness in comparison to sleep reduced the memory performance on high-rewarded memory items. The interaction between sleep/wake and the degree of reward on memory performance was only significant in children. These results show that 12 h of wakefulness can deteriorate the memory performance for high-rewarded representations, whereas sleep can prevent the forgetting of these rewarded representations. It is discussed whether ontogenetic changes in sleep may play a role in conserving relevant but fragile memory representation.

## Introduction

Sleep can support the consolidation of many different memory systems: Adults display better performance for declarative (e.g., words, pictures, and object locations) and non-declarative memories (e.g., finger-tapping sequences, mirror-tracing, or perception) after sleep compared to wake (Rasch and Born, 2013). Moreover, it was shown that sleep fosters the consolidation of emotional memory (Hu et al., 2006; Payne et al., 2008; Wiesner et al., 2015). While declarative memory has been shown to benefit from slow wave sleep (SWS) (Marshall et al., 2006; Mölle and Born, 2011; Ngo et al., 2013), procedural and emotionally toned memories rather benefit from rapid-eye-movement (REM) sleep (Wagner et al., 2001; Nishida et al., 2008; Kirov, 2013; Sopp et al., 2017).

In recent years, it has been shown that sleep fosters the consolidation of reward-associated memory representations as well. In those studies, adults showed better memory performance for high-rewarded or future-relevant than low-rewarded, irrelevant memory items after a daytime nap (Oudiette et al., 2013; Igloi et al., 2015; Studte et al., 2016) or a night of sleep (Wilhelm et al., 2011; van Dongen et al., 2012) compared to wake. For example, van Dongen et al. (2012) asked adult participants to learn word lists. After learning, participants were told that only one of the lists was relevant for later recall. After sleep or wakefulness, however, participants were asked to retrieve both lists after all. While participants in the sleep condition showed an improved memory performance for the relevant words in the wake condition the opposite was true. In another study, Wilhelm et al. (2011) presented word lists to adults. While in some participants expected to retrieve the words at a later point in time, the others did not expect to retrieve them. Only those participants who were told that word lists had a future relevance showed sleep-dependent memory consolidation. These data indicate that subjective relevance moderates sleep-dependent memory processes.

With respect to sleep changes over the course of childhood development, infants and children show a longer duration of sleep (Galland et al., 2012). In the transition to adolescence, the most prominent changes in sleep are decreases in SWS and REM sleep. The basis of these changes in macro sleep architecture is a shift in oscillatory brain activity during sleep: children compared to adults show an increase in EEG-activity in delta (0.5–4 Hz) and theta (4–8 Hz) bands (Kurth et al., 2010; Buchmann et al., 2011; Cirelli and Tononi, 2015). The sleep architecture of adolescents and adults becomes more and more comparable after puberty (Colrain and Baker, 2011; Campbell et al., 2012). Besides the morphology of sleep also its function in memory consolidation develops over the course of childhood (Wilhelm et al., 2012). In a couple of studies it has since been proven that sleep supports the consolidation of declarative memories in children as well (Wilhelm et al., 2012). Backhaus et al. (2008) showed that children remembered word-pair associations better after a night of sleep than after daytime wakefulness. In the same way, the memory performance on an object location task was improved in children after a night of sleep compared to daytime wakefulness (Wilhelm et al., 2008; Maski et al., 2015). Even daytime naps in very young children benefit the memory of object locations (Kurdziel et al., 2013) or the reproduction of observed movements (Konrad et al., 2016). In addition, the consolidation of emotional memory is supported by sleep (Prehn-Kristensen et al., 2009). Here, we observed that children even showed a pronounced sleep-dependent memory bias for emotional pictures compared to adults (Prehn-Kristensen et al., 2013).

Throughout their development, children need to acquire great amounts of knowledge and skills. An early developed reward system (Galvan et al., 2006; Lukie et al., 2014; Mills et al., 2014) is mandatory to determine which of the new experiences are relevant for further, long-term memory consolidation. In a recent study, we showed that sleep benefits the consolidation of reward-related behavior in children (Wiesner et al., 2017). Here, children learned to choose between rewarded and punished stimuli in a probabilistic learning paradigm. After a retention interval containing either sleep (night) or wakefulness (daytime), children were forced to relearn the reward/punishment contingencies. Data showed that sleep stabilized reward-related behavior in healthy children. Whether or not children benefit from sleep with respect to rewarded memory representation, as has been observed in adults before, has not yet been investigated.

The aim of the present study is to investigate whether sleep benefits the maintenance of reward-related memory representations in children more than in adults. Based on the findings that sleep supports reward-related behavior in children (Wiesner et al., 2017) and that sleep supports emotional memories in children more than in adults (Prehn-Kristensen et al., 2013), we hypothesize that children will show lower sleep-dependent rates of forgetting of high-rewarded memory representation than adults.

## Materials and Methods

### Participants

Participants were 16 healthy children (8 females, aged 7–11 years, *M* = 9.7, *SD* = 1.0, *SEM* = 0.3) and 20 healthy adults (10 females, aged 21–29 years, *M* = 25.2, *SD* = 2.3, *SEM* = 0.5) recruited from flyer and advertisements in the local newspapers. According to self-rating, adults did not suffer from psychiatric diseases (Symptom Check List, SCL-90-R; Derogatis, 1992; cut-off: *t* > 67 on any symptom or global scales) or sleep problems (Pittsburgh Sleep Quality Index, PSQI; Buysse et al., 1989; cut-off: 5). Parental ratings revealed that children had no psychiatric symptoms (Child Behavior Check List, CBCL, Achenbach, 1991; cut-off: *t* > 67 on any syndrome scale, on internalizing/externalizing problem scales, or on total score) or sleep problems (Sleep Self Report, SSR-DE; Owens et al., 2000; Schwerdtle et al., 2010; cut-off: 25). Pubertal state was assessed using the pubertal development scale (Watzlawik, 2009): 14 children were pre-pubertal; two girls were just beginning puberty according to their parents. Groups did not differ with respect to IQ (**Table 1**). While all adults were right-handed in the children’s group only 13 of 16 children were right-handed (Edinburgh-Handedness Inventory; Oldfield, 1971). A chi-square test revealed that handedness was not distributed equally across groups (*X* = 4.1, *p* = 0.043, see also **Table 1**). All participants had normal or corrected-to-normal vision.

**Table 1:** Participants’ characteristics and control variables.

	Children (*n* = 16)	Adults (*n* = 20)	Children vs. adults
	
	*M* (*SEM*)	*M* (*SEM*)	*p*-value
Age	9.7 (0.3)	25.2 (0.5)	<0.001
IQ	104 (2.5)	107 (2.1)	0.357
Handedness	13r/3l	20r	0.043
Total sleep time	9.1 (0.14)	7.5 (0.15)	<0.001
Tiredness (0–10)			
Sleep encoding	3.6 (0.54)	4.0 (0.41)	0.573
Sleep retrieval	4.9 (0.66)	3.8 (0.44)	0.182
Wake encoding	3.8 (0.69)	3.1 (0.33)	0.354
Wake retrieval	3.9 (0.62)	3.4 (0.53)	0.561

*M, mean; SEM, standard error of means; r, right-handed; l, left-handed.*

Participants were recruited via newspaper advertisements. All participating adults, children and their parents gave written, informed consent after the procedures had been fully explained. Participants were reimbursed for their participation. The study was approved by the local ethics committee of the medical faculty (reference number: D 525/14).

### Memory Task

**FIGURE 1:**
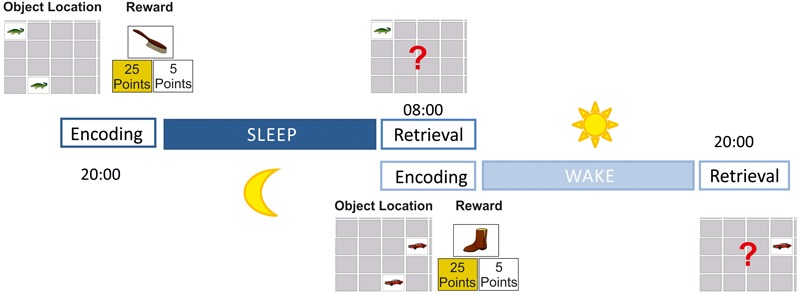
Study design. During encoding participants first learned 18 object locations (learning criterion: 77%) without any cues of allocated reward values; participants then learned to determine which of the object locations were associated with a high and which were associated with a low reward (learning criterion: 100%); at the retrieval session participants were asked to find as many object locations as possible to receive a maximum reward. While encoding was conducted in the evening and retrieval was performed in the morning after sleep in the sleep condition, the encoding session was done in the morning and retrieval in the evening without sleep in between in the wake condition.

The memory task was a 2-D object location task adopted from the well-known card game “Concentration” or “Memory.” In this computer version (created with E-Prime 2.1, Psychological Software Tools, United States) 18 pairs of cards were arranged in six columns and six rows depicting colored cartoon-like pictures. As motifs, we used animals (e.g., a bird, insects, or a bear) and everyday items (e.g., a jacket, a house, a bicycle) on a white background. Stimulus arrangements were presented on a 15.5 inch computer screen (HP ProBook), always centered in the middle of the screen. The screen was always located on a table 50 cm away from the participant. The subtended visual angle of each motif was 4.3°. The encoding sessions were divided into two phases, an object location task and an object reward task (**Figure 1**). The procedure of the object location task has been proven to be sensitive to sleep-dependent memory consolidation in previous studies (Rasch and Born, 2007; Wilhelm et al., 2008, 2011; Diekelmann et al., 2011). This task started by presenting all cards face-up, and the participant was instructed to memorize as many card locations as possible. Here, no time limit was given, and the participant decided when she/he was ready for the next step by pressing a response button. Thereafter, all cards were presented face-down, and one pair after the other was displayed face-up by the computer. A trial started by facing-up the first card of a pair (cue) for 1 s followed by facing-up the second card (target) for 3 s. Then, both cards were faced-down again. After an inter-trial interval (ITI) of 3 s, the next trial started by facing-up the next cue. After all 18 pairs were shown face-up once, the procedure was repeated a second time. Then, an immediate recognition task was conducted. Here, a cue was shown face-up by the computer, and participants were asked to choose the target by using the computer mouse (no time limit). If the choice was correct, a green checkmark appeared for 0.5 s on the chosen position, and the next card was turned over by the computer. If the choice was wrong, then a red X appeared for 0.5 s on the chosen card and the target card’s correct location was displayed. After an ITI of 3 s, the next cue was faced-up. This encoding procedure was repeated until participants made at least 14 correct choices (77%) in one block of trials. Thereafter, the newly developed object reward task began by presenting all cards face-up on the screen: on the left side all high-rewarded motifs (25 points each) were shown, while on the right side all low-rewarded motifs (five points each) were shown. Participants were instructed to memorize the value of each motif (no time limit). In a subsequent memory test, each motif was presented along with two alternative choices (25- or 5-point value, no time limit). The participant was then asked to indicate whether the current motif represented a high- or a low-rewarded item by pressing the corresponding key. If the answer was correct, then a green checkmark appeared on the chosen value for 2 s; the value was then shown again surrounded by a green frame for another 1.5 s. After an ITI of 3 s (blank screen), the next card was presented by the computer. If the choice was wrong, however, then a red X appeared on the chosen value for 2 s, and the correct value was surrounded by a green frame for 1.5 s; then (ITI of 3 s, blank screen) the next card was presented by the computer. This procedure was repeated until participants made 18 correct choices (100%) in one block of trials. In the beginning of the encoding session, all participants were told that they had to memorize as many pairs as possible in order to achieve a high score. Motor performances were done by the dominant hand.

The retrieval session was conducted 12 h after encoding. Participants were presented with the same object location configuration as during encoding; one cue card was displayed face-up and the target card had to be found using the computer mouse. Feedback was given for each trial. After all 18 cue cards were presented once, the retrieval session was finished. There were two equivalent versions with two different sets of pictures and object locations. The assignment of the motifs to the high- and low-reward condition was pseudo-randomized, and the use of picture sets was completely counterbalanced over the experimental conditions. Pretests showed that all motifs were suitable for children and adults; therefore no age-dependent picture sets were created, and all participants worked on the same motifs.

### Procedure

All children and adults participated in two experimental sessions: a sleep and a wake session (**Figure 1**). Both conditions (each being conducted at least 1 week apart) were conducted in the children’s or adult’s home environment to avoid any inconveniences caused by a sleep laboratory environment. Prior to the experimental sessions, all participants were informed about the amount of the reward for good performance. Participants were instructed that their achieved points (25 points for high-rewarded items and 5 points for low-rewarded items) would be summed up at the end of each retrieval session. They also were instructed that a high point score would increase the possibility of receiving the reward. In the adult group, participants were informed that only the top four scorers of all adults would receive an extra bonus of 50€. Children were told that they would receive a small toy (about 2€ each) for good performance in each of both conditions. Children chose their individual present from a “treasure chest” containing 10 different toys (actually all children received both chosen toys independently of their level of performance).

In the sleep condition, the encoding session started between 7 and 8 pm in children and between 8 and 9 pm in adults; after a fixed interval of 12 h, the retrieval task started between 7 and 8 am for children and 8 and 9 am for adults the next morning. In the wake condition, the time schedule was the same but in reverse order; that is, encoding started between 7 and 8 am for children and between 8 and 9 am in adults, and retrieval took place after exactly 12 h the same day in the evening between 7 and 8 pm for children and 8 and 9 pm for adults. The order of sleep-/wake conditions was counterbalanced within groups. During both retention intervals, participants were asked to wear an actigraph recording device affixed on their wrists. Participants were told that these devices were to be worn to record physical activities during the retention intervals. This was done to enhance participants’ compliance to follow the instructions (e.g., not dozing off during the wake condition and going to bed in accordance to their regular sleep-wake cycle during the sleep condition). Since only dummy devices were used, however, no actigraphy data were actually collected.

All participants were asked to rate their current degree of tiredness (one item, 10 cm visual analog scale ranging from not at all to extremely sleepy) at the beginning of the encoding and retrieval sessions.

### Statistical Analysis

To control for differences in encoding performance (see also first paragraph of result section), memory performance was assessed by the relative difference between the object location performance during the last trial of the encoding session and the retrieval session in percent (Wagner et al., 2001; Wilhelm et al., 2011). Here, negative values indicate a loss of memory over the retention interval. To account for the nature of spatial memory and to yield a more continuous measurement for spatial memory, the distance between the correct position and the position indicated by the participant can be used (Rudoy et al., 2009; Deuker et al., 2013; Oudiette et al., 2013). Here, we used a comparable approach: if the correct location was found, then the participant received two points; if the participant chose an immediate neighbor of the correct location, then the participant received one point. All other cases were defined as “failure,” and no points were given. The analysis of memory performance was done by a 2×2×2 ANOVA including the within factors SLEEP (sleep vs. wake), REWARD (high- vs. low-rewarded items) and the between factor AGE (children vs. adults). Comparisons of single means were performed by *t*-tests for dependent samples. According to the Kolmogorov–Smirnov test, the memory performance in children and adults was normally distributed (*p* > 0.149).

## Results

Encoding data revealed that adults performed better than children: adults needed fewer learning trials to reach the learning criterion [children: *M* = 4.4, *SEM* = 0.4; adults: *M* = 2.3, *SEM* = 0.4; *t*(34) = 3.5, *p* = 0.001]. In addition, adults remembered more pair locations correctly at the end of the final learning trial than did children [children: *M* = 14.9, *SEM* = 0.1; adults: *M* = 15.5, *SEM* = 0.2; *t*(34) = 2.3, *p* = 0.028]. The analysis of performance time in minutes revealed that children needed more total time to encode the object locations [children: *M* = 15.5, *SEM* = 1.8; adults: *M* = 9.2, *SEM* = 1.6; *t*(34) = 2.6, *p* < 0.001] as well as to encode the reward contingencies [children: *M* = 9.5, *SEM* = 1.0; adults: *M* = 2.8, *SEM* = 0.4; *t*(34) = 6.6, *p* < 0.001].

**FIGURE 2:**
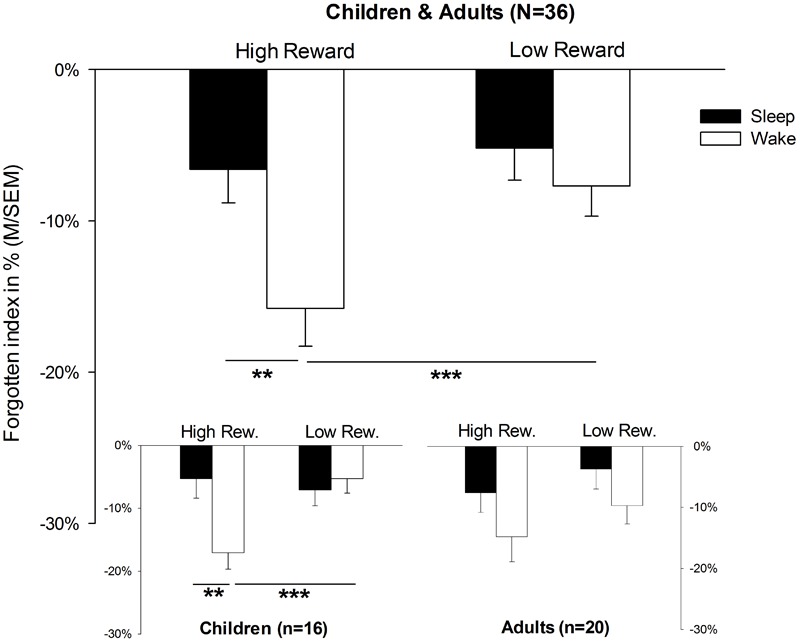
Memory performance. Memory performance in terms of forgetting rates; presented values refer to forgotten object location performance in terms of distance between the correct position and the position indicated by the participant (in %) during the 12 h retention interval either containing sleep or solely wakefulness. Please note that the forgotten index accounts for the distance between the indicated and the correct location. M, mean; SEM, standard error of means; ^∗∗^*p* = 0.01; ^∗∗∗^*p* = 0.001.

The analysis of memory data revealed a main effect for SLEEP, indicating that the rate of forgetting after sleep was lower than after wakefulness in general [*F*(1,34) = 5.8, *p* = 0.022]. Furthermore, the main effect for the factor REWARD [*F*(1,34) = 7.2, *p* = 0.011] suggested that rewarded pairs were more often forgotten than unrewarded pairs (for descriptive statistics, see also **Table 2**). More importantly, however, there was a significant interaction between SLEEP × REWARD [*F*(1,34) = 5.3, *p* = 0.028]. *Post hoc t*-tests for dependent samples showed that rates of forgetting for high-rewarded items was lower in the sleep condition than in the wake condition [*t*(35) = 3.5, *p* = 0.001, **Figure 2**; for descriptive statistics, see **Table 2**]; in the wake condition, rewarded items were more poorly remembered than unrewarded items [*t*(35) = 2.7, *p* = 0.011]. In addition, the interaction SLEEP × REWARD × AGE showed a trend toward significance [*F*(1,34) = 3.7, *p* = 0.062]. A planed decomposition of this latter interaction according to AGE showed that the interaction SLEEP × REWARD still reached significance only in children [*F*(1,15) = 8.2, *p* = 0.012]. A *t*-test for dependent samples revealed that object locations of high-rewarded pairs were forgotten more than low-rewarded pairs in the wake condition [*t*(15) = 3.9, *p* = 0.001]. In the same way, high-rewarded items were forgotten less in the sleep than in the wake condition [sleep: *M* = -5.3, *SEM* = 3.1; wake: *M* = -17.1, *SEM* = 2.6; sleep vs. wake: *t*(15) = 2.9, *p* = 0.010; comparison not shown in **Table 2**]; no other comparison was significant (*p* > 0.5). In adults, all ANOVA effects failed to reach significance (*p* > 0.05). In order to control for a possible influence of handedness on memory performance, we excluded all four left-handed children from the analyses. However, the interaction SLEEP × REWARD still remained significant [*F*(1,12) = 5.5, *p* = 0.037]. In the same way, the comparisons of single means, which reached significance before the exclusion, still remained significant [high vs. low-rewarded pairs in wake condition: *t*(12) = 3.4, *p* = 0.005; wake condition vs. sleep condition for high-rewarded pairs: *t*(12) = 2.2, *p* = 0.047].

**Table 2:** Memory performance.

	Sleep			Wake		
	High rewarded	Low rewarded	High vs. low	High rewarded	Low rewarded	High vs. low
	*M* (*SEM*)	*M* (*SEM*)	*p*-value	*M* (*SEM*)	*M* (*SEM*)	*p*-value
Combined	-6.6 (2.2)	-5.2 (2.1)	0.604	-15.8 (2.5)	-7.7 (2.0)	0.001
Children	-5.3 (3.1)	-7.1 (2.5)	0.661	-17.1 (2.6)	-5.3 (2.3)	0.001
Adults	-7.6 (3.2)	-3.7 (3.3)	0.239	-14.8 (4.1)	-9.7 (3.0)	0.110

*Displayed values (forgotten index) refer to the decrease in object location performance from last trial of encoding to the first trial of retrieval in percent after a 12 h retention interval either containing sleep or solely wakefulness. M, mean; SEM, standard error of means.*

The analysis of tiredness only showed a trend toward a main effect for SLEEP [*F*(1,34) = 3.9, *p* = 0.057], indicating that all participants rated themselves as being slightly more tired in the sleep (*M* = 3.6, *SEM* = 0.3) than in the wake (*M* = 4.1, *SEM* = 0.3) condition. In addition, the trend toward the main effect for SESSION [*F*(1,34) = 3.1, *p* = 0.086], showed that all participants were slightly more tired during the retrieval (*M* = 3.6, *SEM* = 0.3) than during the encoding session (*M* = 4.0, *SEM* = 0.3). However, all other ANOVA effects failed to reach significance (*p* > 0.1). Particularly, the interaction SLEEP × SESSION × GROUP was not significant [*F*(1,34) = 1.5, *p* = 0.235]. According to self-ratings, children displayed longer sleep times than adults [children: *M* = 9.0 h, *SEM* = 0.14; adults: *M* = 7.5, *SEM* = 0.15; children vs. adults: *t*(34) = 7.5, *p* < 0.001]. For descriptive statistics, see **Table 1**.

## Discussion

Here, we observed that wakefulness reduced the memory of relevant information more than sleep did. This was shown by clearly worse memory performance with respect to the high-rewarded condition compared to the low-rewarded condition. In the sleep condition no such remarkable deterioration of memory performance was observed. In addition to this, we found an indication that this selective impact of sleep/wake on relevant memory representations is more pronounced in children than in adults.

The overall rates of forgetting were lower after a period of sleep than after a period of wakefulness. This result points out the beneficial impact of sleep on memory consolidation (Rasch and Born, 2013). In our study, however, this main effect of sleep was clearly driven by the memory performance of high-rewarded items. That is, whereas high-rewarded memory items were forgotten less after sleep than after wakefulness, there was no impact of sleep or wakefulness on the memory for low-rewarded items. At this stage of interpretation one could conclude that sleep had selectively benefited rewarded memory presentations. A somewhat unexpected finding, however, might be the worse overall performance on high-rewarded memory items in comparison to the low-rewarded items. At first glance, one could expect an overall memory benefit for high-rewarded items compared to low-rewarded items (Ito and Lee, 2016; Miendlarzewska et al., 2016); indeed, the opposite was true. Here, two aspects need to be taken into account. Since the rates of forgetting in the sleep condition were rather lower, we suspect that ceiling effects made it impossible to obtain significantly less forgetting for high-rewarded memory items compared to the low-rewarded memory items in the sleep condition. But this can only explain a lack of superior performance with respect to high-rewarded memory items in the sleep condition. More interesting, however, is the finding that wakefulness obviously leads to selective forgetting of high-rewarded memory items. Comparable results were obtained in the study by van Dongen et al. (2012). Using a relatively similar study design, they observed a decrease in memory performance with respect to high-rewarded items in the wake compared to the sleep condition, as we did too. In their study, participants were told to learn two word lists. After the encoding phase, participants were told that only one of the lists was relevant for a later recall. After sleep or wakefulness, however, participants were asked to retrieve both lists. In order to achieve the promised extra bonus for excellent memory performance, participants in our study had to allocate the previously learned object locations (encoded in Round 1) their designated value (encoded in Round 2) retroactively, as well. Importantly, after encoding the reward values (Round 2), participants were not shown the object location matrix again and so they were forced to reactivate the object location matrix from their memory. According to the reconsolidation theory, a reactivation can cause a destabilization of previously learned memory representations which in turn results in worse memory performance (Robertson, 2012; Schwabe et al., 2014; Bonin and De Koninck, 2015; Nader, 2015). Therefore, this selective impact on sleep/wake on the high-rewarded memory items indicates retroactive interference, explaining the memory deterioration of high-rewarded items. Of course, in the sleep condition participants were also forced to attribute the high and low values to the object locations retrospectively. However, in the sleep condition participants were told to go to bed after the encoding session. Therefore subsequent sleep might have initiated stabilization processes (Walker et al., 2003; Diekelmann et al., 2011; Deliens et al., 2013).

The observation that only high but not low-rewarded memory items were affected by sleep/wake emphasizes that the experimental manipulation was successful. Again, comparable results were obtained in the study by van Dongen et al. (2012). In their study, no differences between the sleep and wake condition were found with respect to low-rewarded memory items. These results are also in line with others showing that particularly future-relevant memories are supported by sleep (Wilhelm et al., 2011). Therefore, our data suggest that the lack of sleep/wake-dependent changes in rates of forgetting with respect to the low-relevant condition cannot be interpreted independently of the high-relevant condition. In fact, it appears that a missing impact of sleep on the rates of forgetting of low-rewarded items is present only in the context of high-rewarded items.

In addition to the interaction between the forgetting of high- and low-rewarded object locations during wakefulness and sleep, we found by trend that age had an impact on memory performance: the corresponding comparisons of single means only reached significance only in children. These results point toward a better selective function of sleep in children than in adults. Sleep changes dramatically from childhood to adulthood, not only the total amount of sleep but particularly the slow-wave sleep (SWS) rich in slow-wave activity (SWA), sleep spindle activity, and REM sleep rich in theta activity decrease over the course of puberty (Kurth et al., 2010; Buchmann et al., 2011; Colrain and Baker, 2011; Scholle et al., 2011; Campbell et al., 2012). SWS and particularly SWA are known to drive the consolidation of declarative memory during sleep (Marshall et al., 2006; Mölle and Born, 2011; Ngo et al., 2013). Sleep spindles do not only serve the consolidation of declarative but also procedural memory (Göder et al., 2015; Yordanova et al., 2017a,b). Moreover, REM sleep and theta activity have been associated with a benefit from sleep with respect to the consolidation of emotional as well as procedural memory (Plihal and Born, 1997; Wagner et al., 2001; Nishida et al., 2008; Sopp et al., 2017); meanwhile, it is still under debate whether patterns of REM or non-REM-sleep foster reward-related memory (Perogamvros and Schwartz, 2012; Wiesner et al., 2017). However, we have no reliable information about possible differences in sleep architecture between the children’s and adults’ groups. Whether SWS, SWA, sleep spindles, or even REM sleep preserved the memory for high-rewarded items and were responsible for lower rates of forgetting for high-rewarded items after sleep has yet to be shown in further polysomnographic studies. These data may suggest that sleep has an age-dependent role in stabilizing reward-related behavior. While sleep maintains reward-related associations in children, sleep destabilizes these associations more in adults. From an ontogenetic perspective it could be assumed that children do not yet have many different options of actions, and it would be reasonable during development that sleep stabilizes any reward-related memory representations without deeper evaluation. Both children and adolescents are more sensitive to reward due to a maturational mismatch between an advanced reward-system and a delayed ability for self-control (Galvan et al., 2006; Fareri et al., 2008; Lukie et al., 2014; Mills et al., 2014; Urosevic et al., 2014). In contrast, adults, in fact, have more elaborated options of action so that an immediate stabilization of newly acquired reward-related behavior became less reasonable. This, however, is highly speculative and has yet to be proven. Nevertheless, our data are in line with others indicating that sleep in children has an important function in daytime performance and healthy cognitive development (Dewald et al., 2010; Brand and Kirov, 2011; Astill et al., 2012; Kirov and Brand, 2014).

A limitation of the present study is the relatively small sample size of each age group (16 vs. 20). A higher number of participants might have led to a significant, threefold interaction between the consolidation conditions (sleep vs. wake), the reward conditions (high vs. low), and the age groups. Another limitation is that we did not record sleep parameters. Therefore, all assumptions regarding age-dependent changes in sleep parameters as an explanation for the indicated group-dependent memory performance are over-generalized. Another limitation is the possible ceiling effect in the sleep condition which could explain the lack of differences in rates of forgetting between the high- and low-reward items after sleep. However, it should be noted that the allocation of the card pairs to the high- and low-reward condition was pseudo-randomized and identical for all participants. In addition, the use of both picture sets was counterbalanced across the sleep and wake condition. Since we only observed a decrease in memory performance with respect to high-rewarded items in the wake but not in the sleep condition, the lower memory performance in high-rewarded items cannot be ascribed to a simple effect of item difficulty or other random effects. Another limitation might concern the time of day when experimental sessions were carried out: while encoding in the sleep condition was conducted in the evening, the encoding session of the wake condition was performed in the morning, and the opposite was true for the retrieval sessions. This might lead to confounding between daytime-dependent tiredness and memory performance. Please note, that the subjective tiredness ratings were not different between children and adults with respect to time of day. Therefore, the difference in memory performance between children and adults can hardly be ascribed to simple effects of daytime. Finally, another limitation might be that the subjectively perceived value of the reward could not have been the same for children and adults: While children were told they would receive a small present (less than 2€) for good performance, adults were told that the best five out of 20 participants would receive 50€ each. Obviously, the amount of the reward appears to be higher in adults than in children. If however, the level of reward explains the sleep-dependent impact on high rewarded items, then one could expect adults to outperform children. But the opposite pattern was observed.

Taken together, we observed that high-relevant in contrast to low-relevant memory representations are susceptible to being forgotten after longer periods of wakefulness, and this is probably due to retroactive interferences. Sleep, however, can prevent relevant memory representations from being forgotten. Moreover, the data indicated that this process is more pronounced in children than in adults.

## Author Contributions

AP-K, JS, AP, and CDW designed the study. AB and JS collected the data. AP-K, AB, and JS processed the data and conducted statistical analyses. AP-K drafted the manuscript. AP-K and LB supervised the project. All authors commented on and edited the manuscript drafts.

## Conflict of Interest Statement

The authors declare that the research was conducted in the absence of any commercial or financial relationships that could be construed as a potential conflict of interest.
